# The let-7 family of microRNAs suppresses immune evasion in head and neck squamous cell carcinoma by promoting PD-L1 degradation

**DOI:** 10.1186/s12964-019-0490-8

**Published:** 2019-12-27

**Authors:** Dan Yu, Xueshibojie Liu, Guanghong Han, Yan Liu, Xue Zhao, Di Wang, Xiaomin Bian, Tingting Gu, Lianji Wen

**Affiliations:** 1 Department of Otolaryngology Head and Neck Surgery, the Second Hospital, Jilin University, No. 218, Ziqiang Street, Nanguan District, Changchun, 130041 Jilin Province People’s Republic of China; 2 Department of Oral Geriatrics, School and Hospital of Stomatology, Jilin University, Changchun, 130021 People’s Republic of China

**Keywords:** Head and neck squamous cell carcinoma, Let-7 family, Programmed death-ligand 1, Immune evasion, Glycosylation, TCF-4

## Abstract

**Background:**

Accumulation of immunosuppressive protein programmed death-ligand 1 (PD-L1) has been documented in several cancers and contributes to the evasion of the host immune system. However, cancer cell-intrinsic signaling-dependent control of PD-L1 expression remains to be elucidated. Herein, we aimed to identify the let-7 family of microRNAs as candidates that up-regulate tumor cell PD-L1 expression and mediates immune evasion of head and neck squamous cell carcinoma (HNSCC).

**Methods:**

The expression of let-7 family and PD-L1 was quantified in HNSCC tissues and adjacent normal tissues. PD-L1 degradation was evaluated in HNSCC cells in response to elevated expressions of let-7a or let-7b. The regulation of let-7 family on PD-L1 degradation through a mechanism involving T-cell factor-4 (TCF-4) control of β-catenin/STT3 pathway was evaluated. Immune recognition of HNSCC in vivo was examined in subcutaneous tumor-bearing C3H mice in the presence of let-7a/b and/or CTLA-4 antibody.

**Results:**

The let-7 family were significantly down-regulated in the context of HNSCC, sharing a negative correlation with PD-L1 expression. Glycosylated PD-L1 was detected in HNSCC cells, which was reduced by let-7a/b over-expression. TCF-4, the target of let-7a/b, activated the β-catenin/STT3 pathway and promoted PD-L1 degradation. In vivo analysis demonstrated that let-7a/b over-expression potentiated anticancer immunotherapy by CTLA-4 blockade.

**Conclusions:**

Taken together, our findings highlight targeting let-7 family as a potential strategy to enhance immune checkpoint therapy for HNSCC.

## Background

Head and neck squamous cell carcinoma (HNSCC) is a malignancy that is often manifested as a treatment-resistant tumor arising from the epithelium of the head and neck [1]. As one of the most common cancer around the world, HNSCC is a debilitating cancer with unfavorable patient outcomes and survival rates [2, 3]. In addition, HNSCC exerts a massive burden on the patients’ health and society [4]. Currently, palliative-intent therapy is regarded as a systemic treatment for patients suffering from recurrent and metastatic HNSCC [5]. Sadly, existing literature indicates that the unfavorable prognoses associated with HNSCC have not effectively improved over the past years, highlighting the need for effective treatments for HNSCC [6]. However, recent developments have emphasized immune disorder and immune evasion as key elements implicated in the development and progression of cancers including that of HNSCC [7]. Moreover, another study has suggested the involvement of the let-7 family of noncoding RNAs in the inhibition of the adaptive immune responses and immune evasion in tumors [8].

The Let-7 family of miRNAs plays significant roles in regulating gene expression, participating in a large variety of physiological activities [9]. Previous reports have indicated that miRNAs including let-7 possess the ability to suppress certain diseases such as HNSCC [10]. Programmed death-ligand 1 (PD-L1) is a coinhibitory inducible receptor that usually appears in T-cells and macrophages, with studies flagging the high levels of PD-L1 as a contributory factor in the event of tumor cell immune evasion [11]. More recently, studies have also proposed targeting of the PD-1/PD-L1 axis for immune checkpoint suppression as a therapy for different tumors including HNSCC [12]. The transcription factor 4 (TCF-4) (also ITF2, SEF2 or E2–2) is a commonly widely considered to be a broadly expressed basic helix–loop–helix (bHLH) protein that serves as a homo- or heterodimer [13], with reports highlighting the tumor-suppressive capabilities of TCF-4 in tumors such as the Sonic hedgehog (SHH) medulloblastoma [13, 14]. Moreover, another study identified the presence of a target relationship between let-7 and transcription factor, with evidence suggesting that let-7 targets the lineage-specific transcription factor PLZF in regulating effector function and cell differentiation [15]. Furthermore, accumulation of PD-L1 mediated by STT3 on cancer stem cells has been hypothesized to potentially improve immune evasion in cancers [16], with the Wnt/β-catenin pathway earmarked as a crucial factor in the development of HNSCC [17]. Besides, cytotoxic T-lymphocyte-associated antigen 4 (CTLA-4) has been employed in various immunotherapies, owing to its influence on T cells and ultimately enhancing antitumor capacity [18]. More importantly, CTLA-4 has been reported to be one of the selectively expressed proteins on tumor-infiltrating T cells in HNSCC [19]. However, the interaction between the let-7 family of miRNAs and PD-L1 is yet to be fully realized, thus highlighting the need for deeper investigation. Based on the aforementioned exploration of literature and question to be explored, we subsequently proposed the hypothesis that the let-7 family of miRNAs influences the immune evasion of HNSCC by regulating TCF-4 and PD-L1 via the β-Catenin/STT3 pathway, with the objective of verifying this hypothesis through a series of experiments and ultimately providing enhanced clinical insight for treating HNSCC.

## Methods

### Ethics statement

The current study was conducted with the approval of the Ethics Committee of the Second Hospital, Jilin University. Written informed consent documentations were obtained from all participants prior to the experiment. All animal experiments were performed in strict accordance with the principles and procedures of Guide for the Care and Use of Laboratory Animal by the National Institutes of Health.

### Study subjects

HNSCC tissues and adjacent normal tissues (over 0.5–1 cm away from tumor) were collected from 37 HNSCC patients who had previously undergone continuous surgical resection between January 2011 and December 2013. All patient data was recorded in correspondence with the pathological tissue samples, with a portion of the samples subjected to total mRNA extraction. The collected pathological samples were fixed with neutral buffered formalin, paraffin-embedded and subsequently prepared into sections.

Histopathological diagnoses and classification of HNSCC adhered to the *World Health Organization (WHO) Classification of Tumors of the Digestive System*. In addition, patients with or without lymph nodes/vascular tumor thrombus/perineural invasion/extraesophageal invasion were evaluated, with the clinical data of the patients including gender, age, tumor size and prognostic information confirmed and recorded on a medical record sheet.

### Immunohistochemical staining

The sections were conventionally dewaxed using xylene, dehydrated with gradient alcohol (absolute ethyl alcohol, 95% alcohol, and 75% alcohol, 3 min for each), repaired with citrate repair solution in a pressure cooker, boiled at high temperature for 1.5 min and finally allowed to cool down to room temperature. Next, the sections were rinsed with phosphate buffer saline (PBS), with 50 μL of 3% H_2_O_2_ added to the sections for incubation purposes for a duration of 20 min to eliminate endogenous peroxidase activity. After another PBS rinse, each section was added with programmed death-ligand 1 (PD-L1) (ab213524, Rabbit, Abcam, Shanghai, China) or transcription factor 4 (TCF-4) (ab185736, Rabbit, Abcam, Shanghai, China) for incubation at 4 °C overnight, with normal rabbit serum instead of primary antibody serving as the negative control. After PBS rinsing, each section was incubated with 50 μL polymer reinforcing agent at 37 °C for 20 min, followed by an additional PBS rinse, and incubation with 50 μL enzyme-labeled rabbit anti-polymer at 37 °C for 30 min. Each section was further rinsed twice with PBS (10 min for each), followed by dropwise addition of 100 μL (or 2 drops) of freshly-prepared 3,3′-diaminobenzidine (DAB) for developing. Subsequently, the stained sections were analyzed under a light microscope (CX41-12C02, Olympus Optical Co., Tokyo, Japan), with brown coloration considered to be reflective of a positive staining. Finally, the sections were rinsed with distilled water, counterstained by hematoxylin, dehydrated with gradient alcohol (75% alcohol, 95% alcohol, and absolute ethyl alcohol, 3 min for each), mounted in neutral balsam and observed under a microscope.

The tumor cells presenting with brown-yellow fine particles were regarded as cells with positive expression of PD-L1 and TCF-4. In relation to the positive cells, the cells were scored as followed: positive cells ≤10% was scored as 0 points, 11–51% as 2 points, 51–81% as 3 points, and ≥ 81% as 4 points. Regarding staining intensity, the cells were scored as follows: weak intensity expression was scored as 1 point, moderate intensity expression as 2 points, and high intensity expression as 3 points. The positive cells ≤10% (0 point) were considered negative (−), 3 points were considered weakly positive (+), 4–5 points were regarded as positive (++), and 6–7 points were regarded as strongly positive (+++).

### Cell culture

The 293 T cells (Cell Bank of Chinese Academy of Sciences, Shanghai, China) and the FADU, Cal127 and SCC7 cells (American Type Culture Collection [ATCC], Manassas, VA, USA) were employed for the current study. Initially, the 293 T, FADU and Cal27 cells were cultured with Dulbecco’s modified Eagle’s medium (DMEM) (12800017, Gibco Company, Grand Island, NY, USA) containing 1.5 g/L NaHCO_3_, 10% fetal bovine serum (FBS) and 1% penicillin-streptomycin. Meanwhile, the SCC7 cells were cultured with Royal Park Memorial Institute (RPMI) 1640 medium (31800022, Gibco Company, Grand Island, NY, USA) supplemented with 1.5 g/L NaHCO_3_, 10% FBS and 1% penicillin-streptomycin. The medium was placed on an aseptic bench top, with 5 mL of the medium extracted using a sterile Pasteur tube, which was then added to a 15-mL sterile centrifuge tube. Next, the cells in liquid nitrogen were selected and immersed in a water-bath at 37 °C in order to facilitate rapid thawing. The cells were then extracted using a Pasteur tube and placed in a 15-mL tube, and underwent centrifugation at 800 r/min for 5 min, followed by the removal of supernatant, and resuspension using 1 mL medium. Lastly, the cell suspension was transferred to a 6-cm culture dish and added with 2 mL medium, and then cultured in an incubator at 37 °C with 5% CO_2_ in air. Cell growth was analyzed the following day, with the cell medium renewed every 1–2 days.

### Cell treatment

The core plasmids, LV3 and pBABE (both containing Puromycin resistance gene) over-expressed let-7a, let-7b, Luciferase, flag-Ub and TCF-4 (Cyagen Biosciences Inc. Guangzhou, China) as well as the packaging plasmids pVSVG, pMDL, pREV and PIK were transferred into DH5α competent cells. A plasmid extraction kit (DP103–03, TIANGEN Biotechnology Co. Ltd., Beijing, China) was employed for corresponding plasmid extraction. The extracted plasmids were transfected into 293 T cells using the Turbofect transfection reagent (R0531, Thermo Fisher Scientific, Massachusetts, USA), with the medium changed 12 h later, and medium collection at the 24 h and 48 h time points respectively. The collected medium was filtrated using a 0.22-μm strainer mesh to obtain virus, which was used to infect the HNSCC cell lines. Puromycin (2 μg/mL) was applied in the screening process of the cell lines steadily over-expressing let-7a, let-7b, flag-Ub and TCF-4. G418 (300 μg/mL) was applied to screen the stable cell line containing Luciferase.

### Reverse transcription quantitative polymerase chain reaction (RT-qPCR)

Total RNA content was extracted in strict accordance with the instructions of the Trizol kit (15596–018, Beijing Solarbio Life Sciences Co., Ltd., Beijing, China), followed by RNA concentration determination. According to the instructions of the one-step method miRNA reverse transcription kit (D1801, HaiGene, Harbin, China) and complementary DNA (cDNA) reverse transcription kit (K1622, Beijing Yaanda Biotechnology Co., Ltd., Beijing, China), RNA was reverse transcribed into cDNA, with the reaction conditions as follows: at 42 °C for 30–50 min (reverse transcription) and at 85 °C for 5 s (reverse transcriptase inactivation). The cDNA was diluted to 50 ng/μL and then employed for RT-qPCR. The reaction amplification system was set at 25 μL, with 2 μL added each time. A fluorescent quantitation PCR instrument (ViiA 7, Daangene Co., Ltd. of Sun Yat-Sen University, Guangzhou, China) was applied for RT-qPCR purposes, with the reaction conditions set as follows: pre-denaturation for 4 min at 95 °C, 30 cycles of denaturation for 30 s at 95 °C, annealing for 30 s at 57 °C and extension for 30 s at 72 °C. Two μg of total cDNA was used as a templet, while U6 was regarded as the internal control. The relative expression of the targeted genes was calculated using the 2-^ΔΔCt^ method [20], in which ΔΔCt = ΔCt (experiment group) – ΔCt (control group) and ΔCt = Ct (target genes) – Ct (U6). The experiment was performed in triplicate to obtain the mean value (Table 1).

**Table 1:** Primer sequences for RT-qPCR

Gene	Primer sequence (5′ – 3′)
Let-7a	F: CACCCACCACTGGGAGATAAC
	R: TATGGTTGTTCACGACTCCTTCAC
Let-7b	F: UGAGGUAGUAGGUUGUGUGGUU
	R: CTCAACTGGTGTCGTGGA
Let-7c	F: ACACTCCAGCTGGGTGAGGTAGTA
	R: TGGTGTCGTGGAGTCG
Let-7d	F: GCGGCGGAGAGGTAGTAGGTTG
	R: ATCCAGTGCAGGGTCCGAGG
Let-7e	F: GGGGTGAGGTAGGAGGTTGT
	R: GTGCGTGTCGTGGAGTCG
U6	F: GCTTCGGCAGCACATATACTAAAAT
	R: CGCTTCACGAATTTGCGTGTCAT

Notes: RT-qPCR, reverse transcription quantitative polymerase chain reaction

### PD-L1 glycosylation test

In order to ascertain as to whether PD-L1 was glycosylated, a radio immunoprecipitation assay (RIPA) lysate (P0013B, Beyotime Institute of Biotechnology, Shanghai, China) was applied to lyse FADU and Cal27 cells which were then incubated with PNGase F (G5166-50UN, Sigma-Aldrich Chemical Company, St Louis, MO, USA), and centrifuged at 12000 r/min for to extract the protein content. Next, Western blot analysis was conducted to examine the effects associated with PNGase F on PD-L1. In order to evaluate the degradation of PD-L1 after glycosylation, tunicamycin (654,380, Sigma-Aldrich Chemical Company, St Louis, MO, USA) was employed to treat the cells. Subsequently, 20 μM of cycloheximide (CHX) (HY-12320, MedChemExpress, MedChemExpress, USA) was added to FADU and Cal27 cells to inhibit protein synthesis. Protein content extraction was performed at 0 h, 1 h, 2 h, 4 h, 8 h and 16 h, followed by Western blot analysis to evaluate the expression of PD-L1. Finally, a degradation curve of PD-L1 was plotted [16].

### Peripheral blood monouclear cells (PBMC)-mediated cancer cell killing assay

After mixing interleukin-2 (IL-2) (20 IU ml-1) with PBMCs (STEMCELL Technologies, Vancouver, Canada), HNSCC cells (1 × 10^6^) were co-cultured with PBMCs (1 × 10^7^) for 4 days, followed by isolation of the activated PBMCs using Percoll density gradient centrifugation. The HNSCC cells FADU and Cal27 were labeled with CellTrace (Far Red, Invitrogen, Carlsbad, California, USA), and then cultured with activated PBMCs in a medium (STEMCELL Technologies, Vancouver, Canada) containing human CD3/CD28 tetramer antibody complex in polystyrene tube (FALCON, Canada) for 48 h at 37 °C with 5% CO_2_ in air. Next, the cells were rinsed with PBS, fixed and permeabilized, followed by repair with Fix/Perm solution (BD Biosciences, Franklin Lakes, NJ, USA). The cells were then stained with V450-cleaved caspase 3 antibody (560,627, dilution ratio of 1: 50, BD Biosciences, Franklin Lakes, NJ, USA) and analyzed using BD FACS Canto II (BD Immunocytometry Systems, BD Biosciences, Franklin Lakes, NJ, USA). The HNSCC cells were isolated from the HNSCC/PBMC cell mixture by gating for the CellTrace^+^. Lastly, the activated PBMC-mediated killer HNSCC cells were analyzed by detecting the signal of the V450-cleaved caspase 3 [16].

### Dual-luciferase reporter gene assay

Synthetic TCF-4 3′ untranslated region (UTR) gene fragments were introduced into the renilla luciferase reporter downstream of psiCheck2 vector (Promega, Madison, WI, USA) using the SgfI and PmeI restriction sites. Subsequently, the sequences of the wild type (WT) and mutant type (MUT) in TCF were designed, after which the target fragments of WT and MUT were inserted into the psiCheck2 vector using T4 DNA ligase. Next, the correctly sequenced luciferase reporter plasmids TCF-4 3′-UTR WT and TCF-4 3′-UTR MUT were transfected with let-7a/b into HEK-293 T cells (CRL-1415, Shanghai Xin Yu Biotech Co., Ltd., Shanghai, China). After 48 h, the cells were collected and lysed accordingly. Luciferase detection kits (RG005, Shanghai Beyotime Biotechnology Co., Ltd., Shanghai, China) were employed to detect the relative luciferase activity on Glomax20/20 luminometer fluorescence. Each experiment was performed in triplicate to obtain the mean value [21, 22].

### Western blot analysis

Cells in each group were selected, rinsed twice with PBS, followed by the addition of RIPA lysis buffer (containing phenylmethanesulfonyl fluoride [PMSF] and phosphatase inhibitor) and continual shaking. Next, the cells were centrifuged at 12000 r/min for 30 min at 4 °C, with the supernatant obtained accordingly. After determining the total protein concentration using Bicinchoninic acid assay kits (BCA), 50 μg of proteins were dissolved in 2 × sodium dodecyl sulfate (SDS) loading buffer, boiled at 100 °C for 5 min, separated with 10% sodium dodecyl sulfate-polyacrylamide (SDS-PAGE) and transferred onto a polyvinylidene fluoride (PVDF) membrane using the wet-transfer method. Membrane blockade was then performed for 1 h using 5% skimmed milk powder at room temperature. Next, the membrane was incubated with primary antibodies PD-L1 (ab213524, dilution ratio of 1: 1000, Rabbit), β-Catenin (ab32572, dilution ratio of 1: 1000, Rabbit), T cell factor 4 (TCF-4) (ab185736, dilution ratio of 1: 1000, Rabbit), STT3A (ab242223, dilution ratio of 1: 1000, Rabbit), STT3B (ab122351, dilution ratio of 1: 1000, Rabbit) and β-actin (ab8226, dilution ratio of 1: 1000, Mouse). All the aforementioned antibodies were purchased from Abcam Inc. (Cambridge, UK). After three rinses with tris-buffered saline Tween-20 (TBST), the membrane was incubated with horseradish peroxidase (HRP)-labeled secondary antibody (or goat anti-mouse or goat anti-rabbit, Beijing TransGen Biotech Co., Ltd., Beijing, China) for 1 h. The membrane was subsequently washed again with TBST 3 times, developed using enhanced chemiluminescence (ECL) fluorescence detection kits (BB-3501, Amersham Pharmacia Biotech, Inc. Cambridge, UK) and photographed using the Bio-Rad Image Analysis System (Bio-Rad Laboratories, Hercules, CA, USA). The Quantity One v4.6.2 software was employed to analyze the relative protein expression, which was expressed as the ratio of the gray value of the corresponding protein bands to the β-actin protein band). The experiment was performed in triplicate to obtain the mean value.

### Subcutaneous xenograft models in mice

The C3H mice (aged 6 weeks, weighing 18–22 g) were purchased from Shanghai Lingchang Biotechnology Co., Ltd. Shanghai, China. A total of 1 × 10^6^ SCC7 cells containing Luciferase at the logarithmic phase of growth were resuspended in 200 μL pure DMEM, and then subsequently injected into both the ventral sides of the mice. After tumor formation, all mice were divided into 4 groups based on injection with normal SCC7 cells, SCC7 cells over-expressing let-7a, normal SCC7 cells combined with CTLA-4 antibody (BE0032, Bio X cell, 1 mg/ml) treatment, and SCC7 cells over-expressing let-7a combined with CTLA-4 antibody treatment. The tumor size was measured uniformly every 5 days, with tumor volume = L (length) × W (width)^2^/2. The mice were then injected with D-Luciferin (122799–5, PE, 15 μg/mouse) on the ventral side, anaesthetized using isoflurane and observed accordingly. The tumors were collected 20 days later, and the volume and weight of the tumors were compared and recorded. Tumor dissociation kits (Miltenyi Biotec, Bergisch Gladbach, Germany) and a tumor infiltrating lymphocyte (TIL) gentleMACS dissociation instrument (Miltenyi Biotec, Bergisch Gladbach, Germany) were applied to prepare TIL and the gradient Percoll II liquid (GE Healthcare, Bostan, USA). The density gradient centrifugation method was employed followed by isolation of the TILs from the tumor suspension and flow cytometry analysis. The tumor tissues in TIL without isolation were paraffin-embedded, sectioned and subjected to immunofluorescence assay for PD-L1 expression analysis (64,988, Rabbit, dilution ratio of 1: 200, Cell Signaling, Cell Signaling Technology, Inc., MA, USA), CD8 (ab22378, Rat, dilution ratio of 1: 200, Abcam, Cambridge, USA) and Granzyme b (AF1865, Goat, dilution ratio of 1: 100, R&D Systems, R&D Systems, Minneapolis, MN, USA). Immunohistochemistry was finally applied in order to examine the expression of TCF-4 [16].

### Flow cytometry

The cells were prepared into a single cell suspension and resuspended in staining buffer (BD Biosciences, Franklin Lakes, NJ, USA). FADU and Cal27 cells were treated with APC-PD-L1 (329,708, Mouse, dilution ratio of 1:50, BioLegend, San Diego, CA, USA). The Scc7 cells were treated with BV421-PD-L1 (BioLegend, #124315, Rat. dilution ratio of 1: 100), PerCP-CD3 (100,326, Armenian Hamster, dilution ratio of 1: 100, BioLegend, San Diego, CA, USA), APC/Cy7-CD8a (100,713, Rat, dilution ratio of 1: 100, BioLegend, San Diego, CA, USA), and fixed and permeabilized Pacific blue-IFNγ (505,817, Rat, dilution ratio of 1: 50, BioLegend, San Diego, CA, USA). Lastly, the cells were analyzed using a BD FACS Canto II flow cytometer (BD Immunocytometry Systems, BD Biosciences, Franklin Lakes, NJ, USA) and analyzed with the Flow Jo software [16, 23].

### Statistical analysis

All experimental data were processed using the SPSS 21.0 statistical software (IBM Corp. Armonk, NY, USA). Measurement data were expressed as mean ± standard deviation.

If the data conformed to normal distribution and homogeneity of variance, the paired-designed data between two groups were compared using a paired *t-*test, and unpaired-designed data with unpaired *t-*test. Data between multiple groups were compared by one-way analysis of variance (ANOVA) with a post-hoc test performed using Tukey’s. Data at varying time points between multiple groups were analyzed using repeated ANOVA measures, with post-hoc testing performed using Bonferroni. The correlation among indicators was analyzed by Pearson analysis. A value of *p* < 0.05 was considered to be of statistical significance.

## Results

### The let-7 family of miRNAs is poorly expressed in HNSCC and correlates with PD-L1

**Fig. 1:**
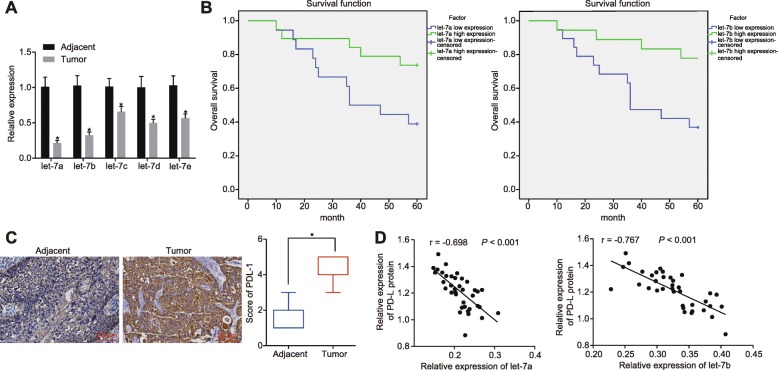
The expression of let-7 family of miRNAs is reduced in HNSCC, and let-7 family of miRNAs is related to PD-L1 expression. **a** the expression of let-7 family of miRNAs in HNSCC examined by RT-qPCR. **b** the relationship between let-7a/7b and prognosis of patients analyzed by Kaplan Meier survival analysis. **c** PD-L1 stains (× 400) in HNSCC tissues and adjacent normal tissues examined by immunohistochemistry. **d** the relationship between the PD-L1 expression in HNSCC examined by western blot analysis and the let-7a/7b expression in HNSCC examined by RT-qPCR. * *p* < 0.05 compared with the control group. The results were measurement data and were expressed as mean ± standard deviation. The results in Fig. 1**a** were analyzed by paired *t* test. *N* = 37. HNSCC, squamous cell carcinoma of the head and neck; RT-qPCR, reverse transcription quantitative polymerase chain reaction; PD-L1, programmed death-ligand 1. Each experiment was conducted at least three times

Existing evidence has suggested that the let-7 family of miRNAs acts as a tumor suppressor and exhibits poor expression levels in numerous tumors [24, 25]. Hence, a series of experiments and investigations were performed to determine the expression of let-7 family of miRNAs in HNSCC tissues. Initially, RT-qPCR was applied to examine the expression of let-7 family of miRNAs in both the HNSCC tissues and adjacent normal tissues, the results of which revealed that the let-7 family of miRNAs was poorly expressed in HNSCC tissues (Fig. 1a; *p* < 0.05), indicating that down-regulated let-7 family of miRNAs could induce the development of HNSCC. In order to examine the relationship between the let-7 family of miRNAs and the life cycle of HNSCC, Kaplan Meier survival analysis (http://kmplot.com/analysis/index.php? P = Service) was performed to further analyze the relationship between let-7a/7b and patient prognosis. By setting the median expression of let-7a/7b in HNSCC tissues as the cut-off threshold to determine the let-7a/7b expression, evidence was obtained indicating that HNSCC patients exhibiting higher expression of let-7a/7b presented with a longer survival time and better survival prognosis compared to that of HNSCC patients with lower expressions of let-7a/7b (Fig. 1b; *p* < 0.05).

As a crucial protein promoting tumor immune evasion, PD-L1, has been demonstrated to exhibit high levels of expression in various tumors. Immunohistochemistry was employed in order to examine the expression of PD-L1 in HNSCC tissues and adjacent normal tissues, with the results revealing that HNSCC tissues presented with higher expression of PD-L1 compared to adjacent normal tissues (Fig. 1c; *p* < 0.05). Next, in order to ascertain as to whether PD-L1 is associated with the let-7 family of miRNAs, Western blot analysis was applied to determine the PD-L1 expression in HNSCC. The combined results of Western blot analysis with the results of RT-qPCR, revealed the existence of a negative correlation between let-7a/7b and the PD-L1 expression (Fig. 1d; *p* < 0.05), ultimately elucidating the relationship between let-7 and PD-L1 in HNSCC. The above results demonstrated that the let-7 family of miRNAs is down-regulated in HNSCC and linked to the expression of PD-L1.

### Let-7a/let-7b of miRNAs inhibits PD-L1 glycosylation and promotes PD-L1 degradation

**Fig. 2:**
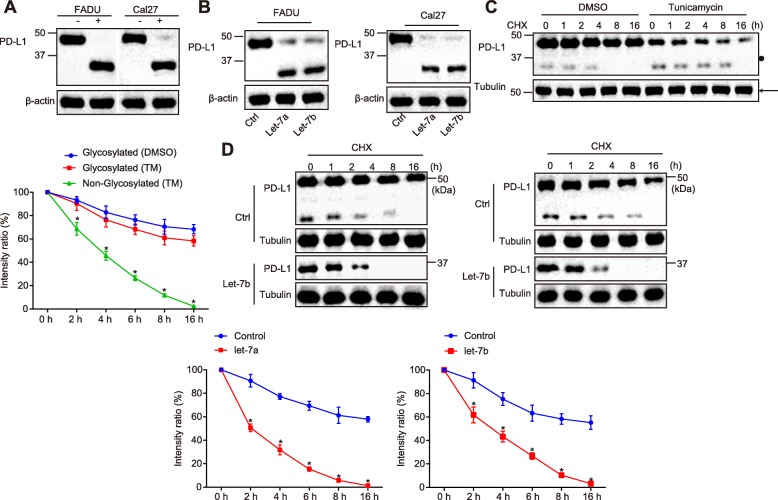
Let-7 represses PD-L1 glycosylation but enhances PD-L1 degradation. **a** the PD-L1 glycosylation modulation in FADU and Cal27 cells examined by addition with PNGase F and western blot analysis. **b**, after overexpression of let-7a and let-7b in FaDu and cal 27, the content and molecular weight of PD-L1 were detected by western blot **c** the degradation rates of PD-L1 was examined by western blot analysis after adding with CHX and tunicamycin, respectively. **d** the degradation rates of PD-L1 was examined by western blot analysis after treatment of over-expressed let-7a and let-7b, respectively. * *p* < 0.05 compared with the control group. The results were measurement data and were expressed as mean ± standard deviation. Data at different time points among multiple groups were analyzed by repeated measure ANOVA, with post hoc test conducted using Bonferroni. The experiment was repeated three times. PD-L1, programmed death-ligand 1; CHX, cycloheximide; IP, immune precipitation; ANOVA, analysis of variance

PD-L1 glycosylation represents a crucial post-translational modified approach that acts to maintain PD-L1 stability and resist degradation. Moreover, PD-L1 has also been documented to be modulated by glycosylation [26]. Therefore, in order to ascertain as to whether PD-L1 was modulated by glycosylation, HNSCC, FADU and Cal27 cells were added with PNGase F, the results of which revealed that the molecular weights of FADU and Cal27 cells exhibited a reduction from 45KD to 33KD following the addition of PNGase F (Fig. 2a), indicating that PD-L1 was modulated by glycosylation in FADU and Cal27 cells. Next, in an attempt to examine whether let-7 regulated the PD-L1 expression, Western blot analysis was performed to determine the PD-L1 expression in FADU and Cal27 cells with over-expressed let-7a/7b. The results revealed that PD-1 expression was significantly decreased, while the molecular weight was found to be 33KD (Fig. 2b). Next, to clarify whether let-7 induced the change in PD-L1 expression by regulating PD-L1 glycosylation, FADU and Cal27 cells were added with CHX and tunicamycin. The results of the Western blot analysis revealed that the degradation rate of PD-L1 was increased at 33KD (Fig. 2c; *p* < 0.05). Lastly, FADU cells treated with over-expressed let-7a/let-7b were added with CHX to examine the degradation rate of PD-L1, and the result indicated that let-7a/let-7b over-expression treatment promoted the degradation rate of PD-L1 (Fig. 2d; *p* < 0.05). The abovementioned results suggested that let-7 suppressed PD-L1 glycosylation and improved PD-L1 degradation.

### Let-7a/let-7b suppresses β-catenin/STT3 pathway by targeting TCF-4

**Fig. 3:**
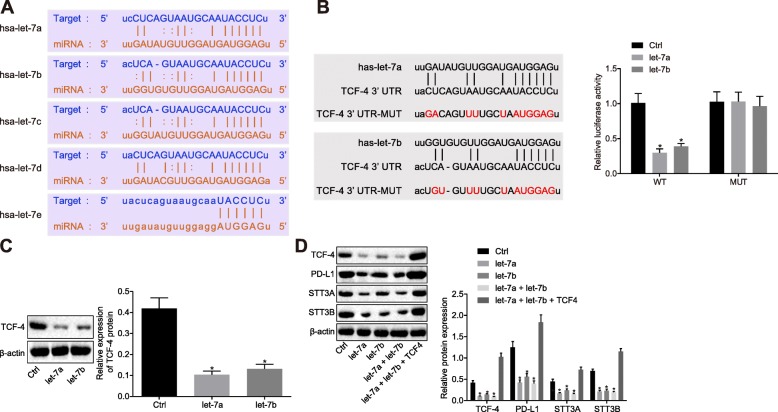
Let-7 family of miRNAs represses β-catenin/STT3 pathway by targeting TCF-4. **a** the binding sequences between let-7 family of miRNAs and TCF-4. **b** the binding of TCF-4 WT/MUT and let-7a/7b examined by dual-luciferase reporter gene assay. **c** the TCF-4 expression in FADU cells treated with over-expressed let-7a/7b examined by western blot analysis. **d** the expression of PD-L1 and β-Catenin/STT3 pathway-related factors in FADU cells after over-expression of let-7a/7b as well as TCF-4 examined by western blot analysis. * *p* < 0.05 compared with the control group. The results were measurement data and were expressed as mean ± standard deviation. Data among multiple groups were analyzed by ANOVA, with Tukey’s post hoc test conducted. The experiment was repeated three times. TCF-4, transcription factor 4; WT, wild type; MUT, mutant type; PD-L1, programmed death-ligand 1; ANOVA, analysis of varianc

In order to investigate the mechanism by which let-7 induces the changes in PD-L1 glycosylation, Starbase data predication was employed, which indicated that TCF-4, the promoter of PD-L1 glycosylation, was the target of the let-7 family of miRNAs (Fig. 3a). Previous literature has further suggested that TCF-4 is essential in the process of β-catenin promoted PD-L1 glycosylation by increasing STT3 transcription [16]. Next, in order to ascertain as to whether let-7a/b regulated the TCF-4 expression, Western blot analysis was applied to examine the TCF-4 expression in FADU and Cal27 cells treated with over-expressed let-7a and let-7b, with the corresponding results revealing that TCF-4 was poorly expressed upon let-7a and let-7b over-expression, suggesting that let-7 affected the expression of TCF-4. Subsequently, to verify whether let-7 binds to TCF-4, a dual-luciferase reporter gene assay was conducted to examine the binding of let-7a/7b and TCF-4, the results of which revealed that let-7a/7b did indeed bind to TCF-4. In addition, the fluorescence intensity was enhanced after the binding site of TCF-4 and let-7a/7b was mutated, indicating that the binding site that TCF-4 interacted with let-7a/7b (Fig. 3b; *p* < 0.05).

Additionally, Western blot analysis was employed to examine the TCF-4 expression in FADU cells treated with over-expressed let-7a and let-7b, revealing a decrease in the TCF-4 expression (Fig. 3c; *p* < 0.05). Next, the expression of β-catenin/STT3 pathway-related factors was detected using Western blot analysis, the results of which revealed that let-7a/let-7b over-expression led to significantly decreased TCF-4, STT3A, STT3B and PD-L1 expression, while exerting no significant effects on β-catenin. Over-expression of let-7a/let-7b as well as TCF-4 led to restoration of STT3A, STT3B and PD-L1 (Fig. 3d; *p* < 0.05), indicating that let-7 could suppress the glycosylation of PD-L1 by regulating TCF-4, thus promoting PD-L1 degradation.

### Over-expression of let-7a/let-7b reduces cell immune tolerance and enhances the cell apoptosis of HNSCC

**Fig. 4:**
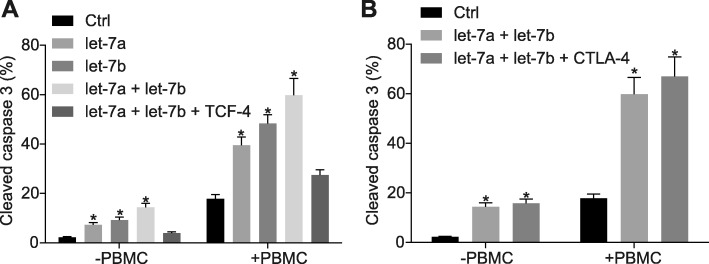
Over-expressed let-7 represses cell immune tolerance but improves the cell apoptosis of HNSCC. **a** the expression of cleaved-caspase3 in FADU cells after treatment with over-expressed let-7a/let-7b/TCF-4 and co-culturing with PBMCs examined by flow cytometry. **b** the expression of cleaved-caspase3 in FADU cells after treatment with over-expressed let-7a/let-7b/TCF-4, co-culturing with PBMCs and combining with CTLA-4 antibody examined by flow cytometry. * *p* < 0.05 compared with the control group. The results were measurement data and were expressed as mean ± standard deviation. Data among multiple groups were analyzed by ANOVA, with Tukey’s post hoc test conducted. The experiment was repeated three times. HNSCC, squamous cell carcinoma of the head and neck; PBMCs, peripheral blood monouclear cells; TCF-4, transcription factor 4; ANOVA, analysis of variance; CTLA-4, cytotoxic T-lymphocyte-associated antigen 4

Next, to explore the effect associated with let-7a/let7b on cell immune tolerance of HNSCC, the FADU cells were labeled with CellTrace, treated with over-expressed let-7a and let-7b, and then co-cultured with activated PBMCs. Subsequent flow cytometry revealed that let-7a/7b over-expression significantly increased the expression of cleaved-caspase3 in FADU and Cal27 cells, while TCF-4 over-expression reversed the above trends (Fig. 4a; *p* < 0.05). Meanwhile, employing a CTLA-4 antibody demonstrated that the over-expression of let-7 combined with the CTLA-4 antibody could effectively up-regulate the expression of cleaved-caspase3, thus improving cell apoptosis in HNSCC (Fig. 4b; *p* < 0.05). The above results suggested that let-7 over-expression suppressed cell immune tolerance and promoted cell apoptosis in HNSCC.

### Over-expression of let-7a/let-7b combined with CTLA-4 antibody treatment inhibits the immune evasion of HNSCC

**Fig. 5:**
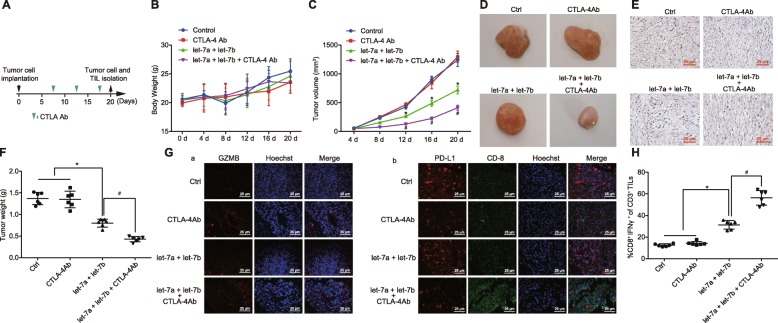
Over-expressed let-7a/7b combined with CTLA-4 antibody suppresses the immune evasion of HNSCC. **a** the tumor cell implantation and the tumor cell and TIL isolation. **b** the body weight of mice at different time points. **c**, the tumor weight in mice at different time points. **d** the tumor sizes in mice after different treatments. **e** the expression of CD3 and TCF-4 in cells of tumors after different treatments (× 400). **f** the weight of isolated tumor of mice after different treatments. **g** the immunofluorescence staining (× 400) of CD8. PD-L1 and GZMB in tumors after different treatments. **h** the percentage of CD8+ and IFN-γ + in CD3+ TILs in tumors examined by flow cytometry. * *p* < 0.05 compared with the control group; # *p* < 0.05 compared with the treatment of let-7a/let-7b. The results were measurement data and were expressed as mean ± standard deviation. Data among multiple groups were analyzed by ANOVA, with Tukey’s post hoc test conducted. Data at different time points among multiple groups were compared by repeated measure ANOVA, with post hoc test conducted using Bonferroni. The experiment was repeated three times. *N* = 6. HNSCC, squamous cell carcinoma of the head and neck; TCF-4, transcription factor 4; PD-L1, programmed death-ligand 1; GZMB, Granzyme B; CD8, cluster of differentiation 8; IFN-γ, interferon–γ; TIL, tumor infiltrating lymphocyte; ANOVA, analysis of variance; CTLA-4, cytotoxic T-lymphocyte-associated antigen 4

In order to explore the specific effects of let-7 on HNSCC, SCC7 cells were subcutaneously injected into C3H mice. Subsequently, the mice were divided into 4 groups based on injection with SCC7 cells, SCC7 cells over-expressing let-7a, SCC7 cells with CTLA-4 antibody treatment, and SCC7 cells over-expressing let-7a combined with CTLA-4 antibody treatment (Fig. 5a). There were no significant differences in the body weight of mice undergoing various treatments (Fig. 5b; *p* > 0.05), while the tumor weight was the lightest among mice treated with SCC7 cells over-expressing let-7a combined with CTLA-4 antibody treatment (Fig. 5c–f; *p* < 0.05). Parts of the tumors were selected, sectioned, and underwent immunofluorescence to detect the contents of PD-L1, CD8 and GZMB in tumors. The obtained results illustrated that after transfection with over-expressed let-7a/b, the expression of GZMB and CD8 was elevated, while the content of PD-L1 was markedly decreased relative to the control group. Compared with let-7a/b over-expression treatment, the treatment with over-expressed let-7a/b combined with CTLA-4 antibody resulted in significantly increased contents of CD8 and GZMB, while there were no evident changes in the expression of PD-L1 (Fig. 5g). Furthermore, immunohistochemistry results demonstrated that treatment of let-7a/b significantly decreased the TCF-4 expression, while treatment of let-7a/7b combined with CTLA-4 antibody resulted in increased content of TILs in CD3+ cells (Fig. 5e). Next, in order to examine TIL activity, TILs were isolated from tumors and subjected to flow cytometry to evaluate the content of IFN-γ in the TILs after undergoing different treatments. The results revealed that the over-expression treatment of let-7a/7b combined with CTLA-4 antibody led to the highest IFN-γ content in TILs (Fig. 5h; *p* < 0.05), indicating that over-expressed let-7a/7b combined with CTLA-4 antibody treatment enhanced the immune activity of TILs, ultimately inhibiting the immune evasion of HNSCC.

### Over-expression of let-7a/let-7b inhibits the TCF-4 expression in HNSCC tissues

**Fig. 6:**
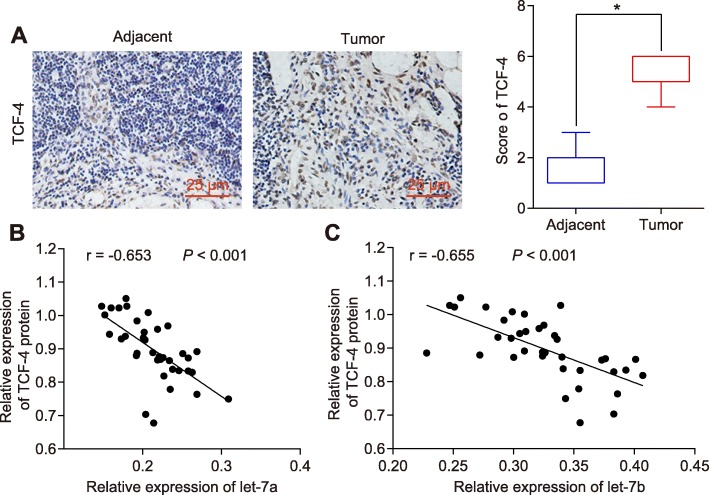
Over-expressed let-7 suppresses the TCF-4 expression in HNSCC. **a** the TCF-4 expression (× 400) in HNSCC tissues and adjacent normal tissues examined by immunohistochemistry. **b** and **c** the correlation between let-7a/7b and TCF-4 by western blot analysis and RT-qPCR. * *p* < 0.05 compared with the control group. The results were measurement data and were expressed as mean ± standard deviation. Data between two groups were analyzed by paired t test. Each experiment was run in triplicate. *N* = 37. TCF-4, transcription factor 4; HNSCC, squamous cell carcinoma of the head and neck; TR-qPCR, reverse transcription quantitative polymerase chain reaction

Additionally, immunohistochemistry was applied in order to examine the TCF-4 expression among both the HNSCC tissues and adjacent normal tissues, with the results demonstrating that HNSCC tissues presented with higher expression levels of TCF-4 compared to adjacent normal tissues (Fig. 6a; *p* < 0.05). Next, Western blot analysis and RT-qPCR were conducted in order to examine the TCF-4 and let-7a/7b expression in HNSCC tissues respectively, in a bid to elucidate the relationship between TCF-4 and let-7. The results revealed that HNSCC patients exhibited high expression of let-7a/7b and low expression of TCF-4, emphasizing the negative correlation between let-7 and TCF-4 (Fig. 6b; *p* < 0.05). Thus, it could be concluded that over-expressed let-7 inhibited the expression of TCF-4 in HNSCC.

## Discussion

At present, the first line treatment approaches for patients suffering from HNSCC include chemotherapy, surgery and radiation therapies [27]. This being said, patients diagnosed with HNSCC generally have unfavorable prognoses with no outstanding effective therapy for the disease, highlighting the need for further investigation and identification of more viable treatment regimens for HNSCC [6]. Immune evasion remains the one of the foremost stumbling blocks affecting the treatment of cancer [28], and furthermore, immune evasion is regarded as a key factor in the occurrence and development of HNSCC [7]. More importantly, studies have also demonstrated the involvement of the let-7 family of miRNAs in exerting therapeutic effects of Rhenium-188-embedded liposomal nanoparticles on orthotopic human head and neck cancer [29]. Additional reports have further implicated the let-7 family of miRNAs in the inhibition of adaptive immune responses and immune evasion associated with various tumors [8]. Besides, the checkpoint inhibitors including anti-PD-L1 in immunotherapy have been previously shown to exert more favorable effects on the treatment of recurrent or metastatic HNSCC [30]. Hence, we asserted the hypothesis that the let-7 family of miRNAs and PD-L1 both participate in the regulation of HNSCC progression. Our key findings demonstrated that over-expression of let-7a/7b acted to suppress the immune evasion of HNSCC cells by targeting TCF-4 and reducing PD-L1 stability via the β-catenin/STT3 pathway.

Our initial results revealed that let-7a/7b was poorly expressed, while PD-L1 and TCF-4 were highly expressed in HNSCC. The expression of PD-L1 has been reported to be upregulated in HNSCC [31]. Similarly, the expression of TCF-21 is also significantly increased in HNSCC [32]. Interestingly, a previous study stated that the expression of let-7 microRNAs was down-regulated in cases involving Salmonella infections [33], while linking the poor expression of let-7 to unfavorable overall survival rates in lung cancer, suggesting that let-7 miRNAs might play a similar role in HNSCC [34]. Additionally, our results verified that let-7 targeted and inhibited TCF-4. In relation to the target relationship between let-7 and transcription factor, previous studies have reported similar mechanisms wherein let-7 targets the zinc finger transcription factor PLAGL2 [35], and indicated that the let-7 targeting of the lineage-specific transcription factor PLZF serves as a regulating effector and influences cell differentiation [15]. Moreover, existing literature has also implicated the down-regulation of the let-7 family of miRNAs and up-regulation of PD-L1 and TCF-4 in the progression of HNSCC, with TCF-4 found to be targeted by let-7, which are all consistent with our findings.

Additionally, we uncovered evidence indicating that over-expression of let-7a/7b promotes PD-L1 degradation and ubiquitination in addition to suppressing the β-Catenin/STT3 pathway. The protein of STT3 is required for the formation of N-glycoside linkage [36]. In addition, a previous study has revealed that the accumulation of PD-L1 mediated by STT3 in cancer stem cells enhances immune evasion associated with certain types of cancers [16]. Moreover, we identified that over-expression of let-7a/7b suppressed immune tolerance, while over-expression of let-7 combined with CTLA-4 inhibited immune evasion, and significantly increased the contents of CD8 and GZMB, as well as the content of TILs in CD3+ cells, in addition to higher levels of IFN-γ in TILs. Elevated expression of infiltrating T-cell subsets including CD3+ cells has also been shown to be correlated with more favorable prognoses in colon cancer patients [37]. Similarly, TILs have been identified as indicators of favorable prognosis and reaction to treatment in various types of cancer, with higher expression levels of prognostic biomarkers (CD3+ TILs, CD4 + TILs and CD8+ TILs) also considered to indicative of improved overall survival in cases of human papillomavirus [38]. Furthermore, the let-7 family of miRNAs has been demonstrated to exercise significant function in shrimp immune response of anti-viral infection [39]. As well, the combined treatment of α-CTLA-4 and α-PD-L1 antibody blockade has been shown to confer protection against immune evasion and aid in the control of metastatic osteosarcoma [40]. All in all, the aforementioned findings illustrate similar trends with existing literature.

## Conclusions

**Fig. 7:**
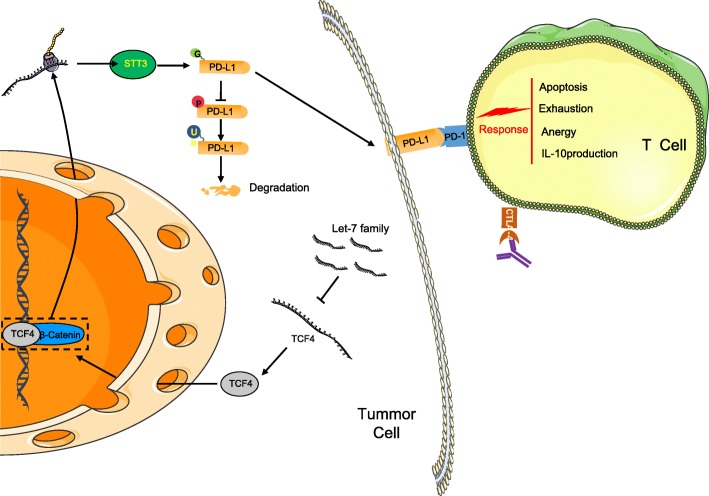
The molecular mechanism involved in let-7 family of miRNAs regulating the immune evasion of HNSCC by mediating TCF-4, β-Catenin/STT3 pathway and PD-L1. Let-7 family of miRNAs inhibited the expression of TCF-4, suppressed β-Catenin/STT3-mediated PD-L1 glycosylation, reduced PD-L1 stability, promoted the ubiquitination and degradation of PD-L1, and improved the recognition ability of T cells to HNSCC cells. miRNAs, microRNAs; HNSCC, squamous cell carcinoma of the head and neck; TCF-4, transcription factor 4; PD-L1, programmed death-ligand 1

In conclusion, the key findings of the current study highlight the anti-oncogenic effect of the let-7 family of miRNAs on the progression of HNSCC. This study revealed that over-expression of let-7 family of miRNAs inhibited the immune evasion in HNSCC by suppressing the expression of TCF-4, β-Catenin/STT3-mediated PD-L1 glycosylation and PD-L1 stability, while promoting the ubiquitination and degradation of PD-L1, in addition to the recognition ability of T cells to HNSCC cells (Fig. 7). Our findings provide a novel insight in relation to potential therapeutic strategies for HNSCC. Nevertheless, it should also be pointed out that the specific mechanism involving let-7, PD-L1 and TCF-4 was not fully investigated and thus requires further research to fully realize their therapeutic effects for HNSCC.

## Data Availability

The datasets generated/analysed during the current study are available.
